# Investigating the Structural Impacts of I64T and P311S Mutations in APE1-DNA Complex: A Molecular Dynamics Approach

**DOI:** 10.1371/journal.pone.0031677

**Published:** 2012-02-27

**Authors:** C. George Priya Doss, N. NagaSundaram

**Affiliations:** Centre for Nanobiotechnology, Medical Biotechnology Division, School of Biosciences and Technology, VIT University, Vellore, Tamil Nadu, India; Università di Napoli Federico II, Italy

## Abstract

**Background:**

Elucidating the molecular dynamic behavior of Protein-DNA complex upon mutation is crucial in current genomics. Molecular dynamics approach reveals the changes on incorporation of variants that dictate the structure and function of Protein-DNA complexes. Deleterious mutations in APE1 protein modify the physicochemical property of amino acids that affect the protein stability and dynamic behavior. Further, these mutations disrupt the binding sites and prohibit the protein to form complexes with its interacting DNA.

**Principal Findings:**

In this study, we developed a rapid and cost-effective method to analyze variants in *APE1* gene that are associated with disease susceptibility and evaluated their impacts on APE1-DNA complex dynamic behavior. Initially, two different *in silico* approaches were used to identify deleterious variants in *APE1* gene. Deleterious scores that overlap in these approaches were taken in concern and based on it, two nsSNPs with IDs rs61730854 (I64T) and rs1803120 (P311S) were taken further for structural analysis.

**Significance:**

Different parameters such as RMSD, RMSF, salt bridge, H-bonds and SASA applied in Molecular dynamic study reveals that predicted deleterious variants I64T and P311S alters the structure as well as affect the stability of APE1-DNA interacting functions. This study addresses such new methods for validating functional polymorphisms of human *APE1* which is critically involved in causing deficit in repair capacity, which in turn leads to genetic instability and carcinogenesis.

## Introduction

Base Excision Repair (BER) is one of the DNA repair systems which are essential for the maintenance of genome integrity. Major genes involved in the BER pathway include *MUTYH*, *OGG1*, *APEX1*, *XRCC1* and *PARP1* [1]. Key enzyme involved in BER pathway is A-purinic endonuclease-1 (APE1), which cleaves the DNA sugar-phosphate backbone at a position 5′ of AP (apurinic/apyrimidinic) [2] sites to prime DNA repair complex synthesis. AP sites arise frequently in normal DNA from a variety of causes: exposure to endogenously produced reactive oxygen species, ionizing radiation, and alkylating agents. AP sites are pre-mutagenic lesions that can prevent normal DNA replication and transcription. APE1 electrostatically orients a rigid, pre-formed DNA-binding face and penetrates the DNA helix from both the major and minor grooves, stabilizing an extra-helical conformation for target abasic nucleotides. APE1-DNA contacts facilitate and stabilize the extra-helical AP site [3]. Involvement of DNA repair genes such as *APE1* along with environmental exposures has paved a way to identify polymorphic variants that have the potential to cause cancer risk [4]. Polymorphisms in *APE1* result in conformational change of the protein, which in turn affects regular interaction with DNA. Altered protein function causes deficit in repair capacity, which may lead to genetic instability and carcinogenesis [5]–[8]. Single nucleotide polymorphisms (SNPs) are the most common and simplest form of human genetic variants. Within coding regions; non-synonymous SNPs (nsSNPs) might change the physicochemical property of a wild type amino acid that affects the protein stability and dynamics, disrupts the interacting interface, and prohibits the protein to form a complex with its partners [9]–[12]. Identification of nsSNPs responsible for specific phenotypic variation to be a major concern that is very difficult to solve, requires multiple testing of hundreds or thousands of SNPs in the candidate genes [13], [14]. In this aid, experimental based approaches were used to identify polymorphisms in *APE1* gene and their disease associations were discussed extensively. The current limitations in the experimental based approaches such as cost and time emerged the use of *in silico* methods and specifically molecular dynamics simulations in SNP analysis. Recently, few *in silico* studies were carried out in identifying the possible functional and deleterious nsSNPs in *APE1* gene [15]–[16]. Several *in silico* methods have been developed recently, whose goal is to extrapolate the functional and deleterious nsSNPs from the neutral ones based on sequence and structure based approach [17]–[23]. These methods utilize information such as physicochemical properties [24], protein structure, and cross species conservation [25] to predict deleterious nsSNPs. The structure of a protein can change in various ways due to the biochemical differences of the amino acid variant (acidic, basic, or hydrophobic), and by the location of the variant in the protein sequence (by affecting tertiary or quaternary structure or the active site where substrate binds).

Current interest in molecular genetics is focused on disease-gene association that is, identifying which DNA variation or a set of DNA variation is highly associated with a specific disease at structural level. Knowledge of the three dimensional (3D) structure of a gene product will aid in understanding the function within the cell and its role in causing disease. Proteins with mutations do not always have the 3D structures that are analyzed and deposited in Protein Data Bank (PDB). Therefore, it is necessary to construct 3D models by locating the mutation positions and the residues in 3D structures. This is a simple way of detecting what kind of adverse effects that a mutation can have on a protein at structural level. It has been understood Protein-DNA complexes play multiple important roles in cellular processes that involve DNA transactions, such as transcription, replication, recombination, and DNA repair as well as the packaging of chromosomal DNA [26]. Proteins that interact with DNA in these complexes either serve as enzymes to catalyze biochemical reactions [27] or simply act as an “architectural scaffold” [28] to manipulate the structure of DNA by, for example, bending and/or wrapping DNA. Involvement of SNPs in these Protein-DNA complexes at molecular level is impossible. The main goal of *in silico* analysis presented in this work is to develop molecular dynamics simulation methodology that provides the direct link between deleterious nsSNPs at both functional and structural level. In this present study, we focused on variants in *APE1* gene associate with disease susceptibility and their impacts on APE1-DNA complex dynamic behavior. The scope of this work is twofold. On one hand, it addresses the application of publicly available *in silico* tools such as Sorting Intolerant From Tolerant (SIFT) [21], Hidden Markov Model (HMM) based Protein analysis through evolutionary relationship (PANTHER) [22], Polymorphism Phenotyping (PolyPhen) [13] and I-mutant [23] to identify deleterious nsSNPs in *APE1* gene. On the other hand, structural analysis was carried out to know, how the APE1-DNA interacting function gets affected with respect to the identified deleterious nsSNPs in *APE1* through Molecular Dynamic (MD) approach using GROningen MAchine for Chemical Simulations (GROMACS). In this study, investigations of structural consequences of these novel *APE1* mutations I64T and P311S were explored using molecular dynamics simulations. Separate simulations were conducted to investigate the conformational behavior of the APE1-DNA complex in two different states namely, native and mutant.

## Results

### SNP information from database

The data set for analysis of potential impact of polymorphism in *APE1* gene investigated in this work was retrieved from dbSNP database [29]. For human *APE1* gene a total of 59 SNPs were found in database, out of that 10 (0.17%) were nsSNPs, 11 (0.19%) occur in the mRNA 3′UTR, 14 (0.23%) occur in the mRNA 5′UTR, and (0.17%) occur in intronic region. We selected nsSNPs for our investigation. We applied our method to evaluate the *APE1* gene association with disease, and its related information for biomedical literature was taken from OMIM (Online Mendelian Inheritance In Man) [30]. Experimental related information about SNPs of *APE1* gene was obtained from Swiss-Prot database [31]. Out of ten, seven nsSNPs exhibited transition (A→G, C→T), while remaining three exhibited transversion (A→C, G→T, and C→T). Co-ordinates file of APE1 Protein-DNA complex with PDB ID 1DE8 was obtained from PDB for structural analysis using Molecular dynamics approach.

### Impact of deleterious nsSNPs in *APE1* gene

The functional impact of *APE1* nsSNPs can be assessed by evaluating the importance of the amino acids they affect. Identifying the nsSNPs conferring susceptibility or resistance to common human diseases should become increasingly feasible with improved *in silico* tools. In this analysis, we employed four *in silico* tools for determining the functional significance of ten nsSNPs in *APE1* gene. Table 1 presents distribution of the deleterious and neutral nsSNPs in *APE1* gene by these *in silico* methods. SIFT predicts whether an amino acid substitution affects the protein function based on sequence homology and the physical properties of amino acid. This tool calculates a score for every substitution and predicts the functional effect. Three nsSNPs with dbSNP ID rs61730854, rs33956927 and rs1803120 having a tolerance index score of 0.00, 0.02 and 0.00 respectively were identified to be deleterious by SIFT. Rest of the seven nsSNPs were predicted to be tolerant by SIFT exhibiting tolerance index score range of 0.006–1.00. We further analyzed these ten nsSNPs using PolyPhen based on empirically derived rules. PolyPhen uses information about the structure of the protein (hydrophobicity, charge effects, and changes in molecular contacts), available structure data from Protein Data Bank, and multiple sequence alignment. Based on Position Specific Independent Count (PSIC) score difference, variant is predicted to be probably damaging or possibly damaging or benign. However the multiple sequence alignment is generated by PSIC software which assigns a score that indicates the probability of a given amino acid occurring at a particular position against any random position. Based on the calculated alignment score and differences in structural parameters, the PolyPhen designates the mutation as being “benign”, “possibly damaging”, and “probably damaging” with the respective scores of <1.5, >1.5 and >2.0 respectively. Two nsSNPs with an IDs rs61730854, and rs1803120 having score 2.286, and 3.123 were predicted to be probably damaging the protein structure and function. While three nsSNPs with IDs rs34632023, rs2307486, and rs1048945 showed a score 1.734, 1.811 and 1.539 were designated as possibly damaging to protein function. Remaining five nsSNPs with a score range of 0.465 to 1.272 were designated as benign by PolyPhen. To validate the prediction of SIFT scores, we used HMM based evolutionary approach PANTHER to validate its impact on protein function upon single point mutation. Out of these ten nsSNPs taken for our analysis, three nsSNPs rs61730854, rs1803120 and rs1803118 are having score −5.56072, −7.92488 and −3.68399 were designated as deleterious. Other seven nsSNPs exhibited a subPSEC score range of −1.63595 to −2.93478. In order to improve prediction accuracy of structure based approach tools, we used support vector machine based tool I-Mutant 2.0. The protein stability change due to single point mutation was predicted using I-Mutant 2.0. A score less than “0” means the mutation decreases the stability. The smaller, the score more, the more confident is the prediction. Conversely, a score more than “0” means mutation increase the protein stability. Seven nsSNPs with dbSNP IDs rs33956927, rs2307486, rs1803120, rs1803118, rs1048945, rs61730854, rs113056798 showed the negative DDG value −0.10, −1.13, −1.00, −0.33, −1.73, −0.01, −0.62 Kcal/mol, were considered to be least stable and deleterious. By comparing the prediction scores of all the four *in silico* tools, nsSNPs with IDs rs61730854 (I64T) and rs1803120 (P311S) were designated as highly deleterious and functionally significant. The I64T polymorphism is the result of nucleotide change from C to T and results in the substitution of hydrophilic threonine for hydrophobic isoleucine at amino acid 64 of *APE1* gene, thus affecting hydrophobic interactions. P311S involves change of non polar hydrophilic residue proline to polar neutral residue serine. It is well-known that during folding of the polypeptide chain, the amino acids with a polar (hydrophilic) side chains are found on the surface of the molecule, while amino acids with non polar (hydrophobic) side chains are buried in the interior. Thus, this variation may lead to change in the folding pattern of the protein.

**Table 1:** List of nsSNPs showing deleterious/non- deleterious scores by SIFT, PANTHER, PolyPhen and I-Mutant.

rs IDs	Alleles	AA Position	Toleranceindex	PredictedImpact	subPSEC score	PSIC score	Predicted impact	DDGKcal/mol	Predicted impact
rs113056798	A/G	T313A	0.31	Tolerant	−2.93478	0.629	Benign	**−0.10**	**Decrease Stability**
rs61757709	A/C	K35Q	0.08	Tolerant	−1.80353	1.272	Benign	1.58	Increase Stability
**rs61730854**	**C/T**	**I64T**	**0.00**	**Intolerant**	**−5.56072**	**2.286**	**Probably Damaging**	**−1.13**	**Decrease Stability**
rs34632023	C/T	G39E	0.99	Tolerant	−1.63595	**1.734**	**Probably Damaging**	1.97	Increase Stability
rs33956927	C/T	G241R	**0.02**	**Intolerant**	−2.15336	0.598	Benign	−1.00	Decrease Stability
rs2307486	A/G	I64V	0.06	Tolerant	−2.62028	1.811	**Probably Damaging**	−0.33	Decrease Stability
**rs1803120**	**C/T**	**P311S**	**0.00**	**Intolerant**	**−7.92488**	**3.123**	**Probably Damaging**	**−1.73**	**Decrease Stability**
rs1803118	C/T	A317 V	0.27	Tolerant	**−3.68399**	0.761	Benign	−0.01	Decrease Stability
rs1130409	G/T	D148E	1.00	Tolerant	−2.25414	0.465	Benign	0.17	Increase Stability
rs1048945	C/G	Q51H	0.09	Tolerant	−1.80925	**1.539**	**Probably Damaging**	−0.62	Decrease Stability

Highly deleterious by SIFT, deleterious subPSEC score by PANTHER, Probably and possibly damaging by PolyPhen and Negative score of I-Mutant were highlighted in bold.

### Modeling and analysis of local environment changes

**Figure 1:**
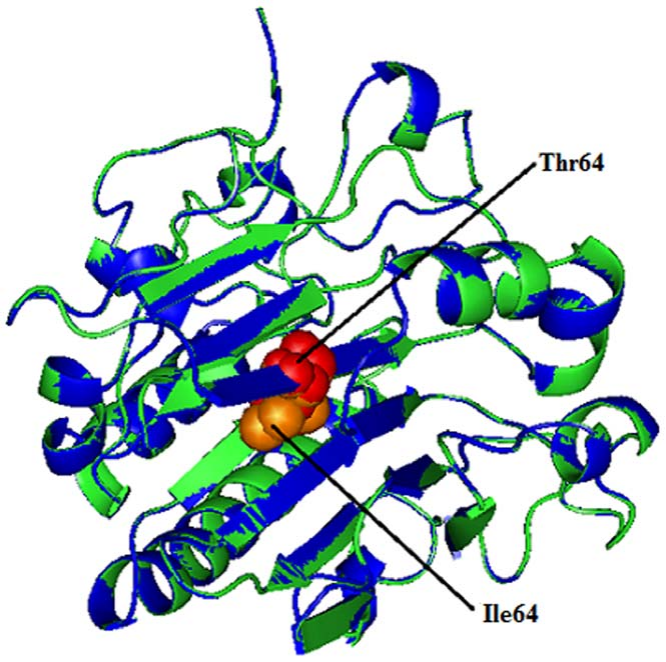
Superimposed structure of native amino acid isoleucine in sphere shape (orange color) with mutant amino acid threonine (red color) at position 64 in PDB ID 1DE8 of *APE1*gene.

**Figure 2:**
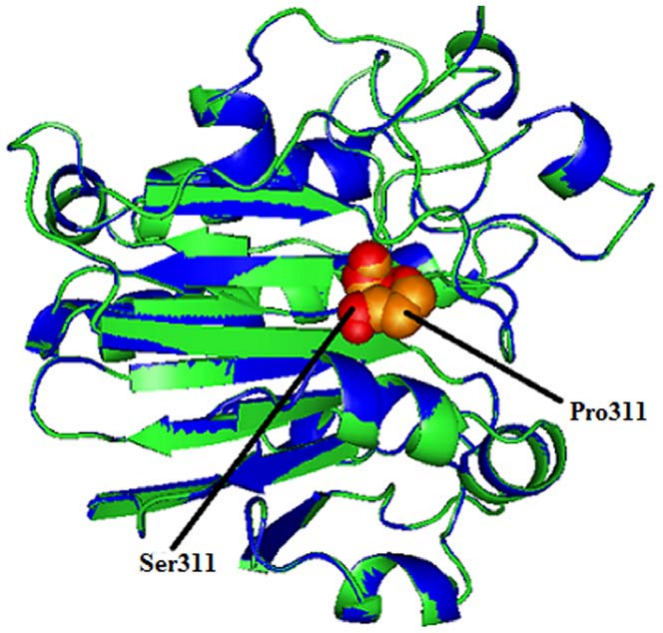
Superimposed structure of native amino acid proline in sphere shape (orange color) with mutant amino acid serine (red color) at position 311 in PDB ID 1DE8 of *APE1* gene.

**Figure 3:**
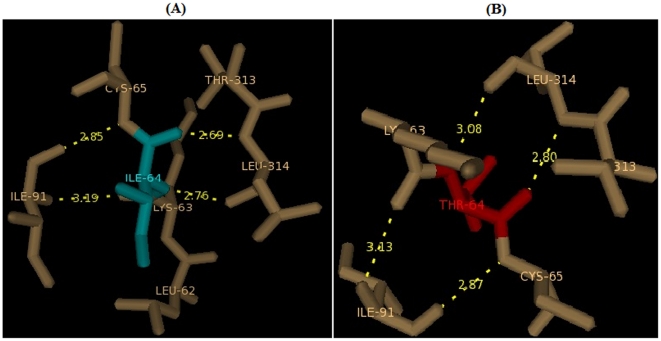
Changes in local environment and polar residue distance of APE1 protein brought about by an I64T mutation. (**A**) The native type isoleucine residue (cyan) shows distance with surrounding residues.(**B**) Substitution of I64 residue to threnioine (red) shows surrounding residues and polar distance changes.

**Figure 4:**
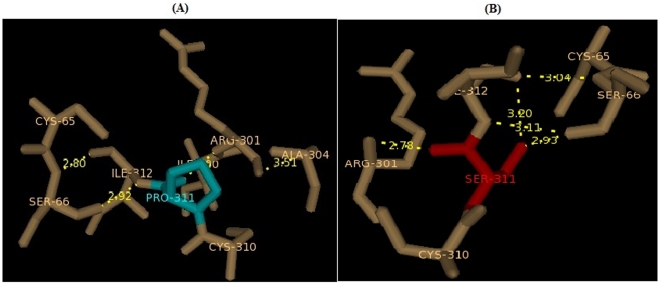
Changes in local environment and polar residue distance of APE1 protein brought about by P311S mutation. (**A**) The native type proline residue (cyan) shows distance with surrounding residues. (**B**) Substitution of P311 residue to serine (red) shows surrounding residues and polar distance changes.

Modeling of predicted deleterious nsSNPs I64T and P311S on protein structure with PDB ID 1DE8 was performed using Swiss-PDB Viewer [32]. Super imposed structures of native and mutant models were shown in Figures 1 and 2. Within the range of 4 Å from the mutational point, surrounding residue changes and polar contact between residues were analyzed. It was observed through PyMOL [33] and shown in Figures 3 and 4. Substitution of hydrophobic residue isoleucine to hydrophilic residue threonine (I64T) leads to hydrophobic change at the core of the protein that could result in the destabilization of the β-sheets. The drift in hydrophobic to hydrophilic property can in result in loss of hydrogen bonds and disturbs correct folding. This mutation can disturb interactions with other molecules or other parts of the protein. In Figure 3A illustrates the distance between native I64, neighboring residues T313 and L314. The native I64 maintains the distance range of 2.69 Å and 2.76 Å between neighboring residues T313 and L314 respectively, while in mutant model substitution of threonine, Figure 3B illustrates the distance between mutant 64T and its neighboring residues T313 and L314. Substitution of threonine increases the distance of T313 to 2.80 Å and increase the distance of L314 to 3.08 Å. In P311S polymorphism there is change in drift of charge from non-polar to polar residue. P311 substituted with hydrophilic serine leads to change in the surrounding amino acids and their formal distance. From Figure 4A, it was observed that native P311 could not make any contact with surrounding residues, while substituted serine Figure 4B makes contact with R301 and maintained a distance of 2.78 Å. The substitution of serine at 311 introduces a change in charge at the core and could introduce repulsive interactions between neighboring residues. Mutation results, changes in local environment of APE1 amino acid residues and enhance in the modification of polar residues distances. These nsSNPs I64T, P311S leads to decrease the stability of protein as predicted by I-Mutant 2.0: thus must be having conformational changes at the structural level.

### Molecular dynamics conformational flexibility and stability analysis

**Figure 5:**
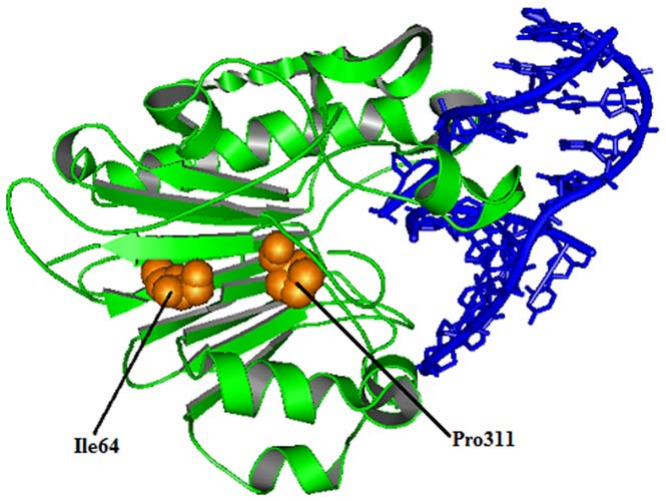
Co-crystal structure of APE1 protein with DNA and native amino acid isoleucine and proline at position 64 and 311 respectively.

**Figure 6:**
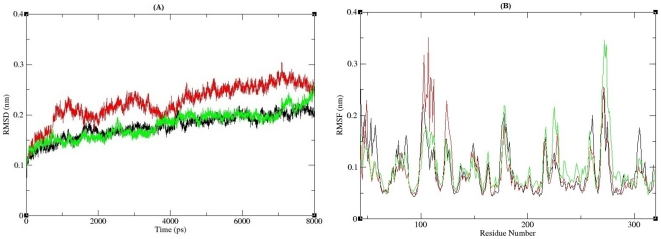
RMSD and RMSF analysis of APE1-DNA complexes. (**A**) Backbone RMSD of the APE1-DNA complexes. The ordinate is RMSD (nm), and the abscissa is time (ps). Black, Red and Green lines indicate native, I64T and P311S structures respectively. (**B**) RMSF of the carbon alpha atoms over the entire simulation. The ordinate is RMSF (nm), and the abscissa is residues. Black, Red and Green lines indicate native, I64T and P311S structures respectively.

Molecular dynamics simulation approaches pay the way for in depth analysis on structural effects upon mutation in APE1-DNA complex. The DNA strands in the co-crystal with APE1 (Figure 5) has a 30 degree bend at the AP site. Based on this, we presumed that I64T and P311S, would affect the angle of interaction, and it was investigated through subsequent molecular dynamics approach. Potential energy for native and mutant proteins in bound (Protein-DNA) and unbound states (protein) were calculated. They exhibited a range from −7.94830 to −8.87568 kJ/mol in bound state; while in unbound state range is from −5.53928 to −5.64950 kJ/mol (Table 2). The deviation between native and mutant structures were evaluated by their root mean square deviation (RMSD) values which could affect protein stability. We calculated the RMSD for all the atoms from the initial structure, which were considered as a central criterion to measure the convergence of the protein system concerned. The backbone RMSDs were calculated for the trajectories of three complexes i.e., native modeled complex of 1DE8 with substitution of threonine at position 64 and modeled complex of 1DE8 with substitution of serine at position 311 from the starting structures as a function of time. The root mean square deviation (RMSD) of the backbone atoms relative to the corresponding starting structures was calculated and the results are reported in Figure 6A. For all the three structures a considerable structural changes was observed during the initial few picoseconds leading to a RMSD to ∼0.15 nm, followed by a notable structural deviations for the rest of simulations. The RMSD reach a stable value of ∼0.15 nm within the range of ∼4–6.5 ns for native and mutant P311S structure. On other hand, I64T attained stable value of ∼0.25 nm within the range of ∼4–6.5 ns. The final RMSD value of ∼0.2–0.25 nm for all three simulations was observed. The I64T retained maximum deviation till end and around the period of 7,000 ps it attains RMSD value of ∼0.275 nm. A small difference between the average RMSD values after the relaxation period (∼0.15 nm), lead to the conclusion that the constant ranges of deviation in the native and mutant structures reflect that mutation could affect the dynamic behavior of mutant complexes. From their starting structure, P311S showed minimum deviation but I64T deviated maximum over the native structure. In order to evaluate the conformational fluctuation between native and I64T, and P311S root mean square fluctuations (RMSFs) was generated from trajectory analysis obtained by molecular dynamic simulation. The RMSF value of carbon alpha of each amino acid residue was calculated from the trajectory data of the native, I64T and P311S as shown in Figure 6B. The RMSF calculation shows that, in the entire simulation period native complex residues fluctuate within the range of ∼0.05–0.2 nm. I64T attained maximum level of fluctuation up to ∼0.3 nm in the residue range of ∼100–130 and P311S shows the same level of fluctuation in the residue range of ∼260–270. Consistent with the RMSD analysis, RMSF of I64T and P311S was notably deviated from native structure in the whole simulation. Deviation in the flexibility of I64T and P311S mutants are further analyzed by validating the number of hydrogen bonds formed between APE1 protein and its binding DNA.

**Table 2:** Potential energy of APE1 protein native and mutant models in bound and unbound states.

rs IDs	APE1 native and mutant models	Bound statekJ/mol	Unbound statekJ/mol
	Native	−8.87568	−5.64950
rs113056798	T313A	−8.78562	−5.60148
rs61730854	I64T	−7.94830	−5.55177
rs33956927	G241R	−8.39492	−5.60224
rs2307486	I64V	−8.68980	−5.56422
rs1803120	P311S	−8.17593	−5.61756
rs1803118	A317 V	−8.56784	−5.56217
rs1130409	D148E	−8.63908	−5.53928
rs1048945	Q51H	−8.25221	−5.58748

### Effect of mutations I64T and P311S in hydrogen bonds, salt bridges and solvent accessible surface area

**Figure 7:**
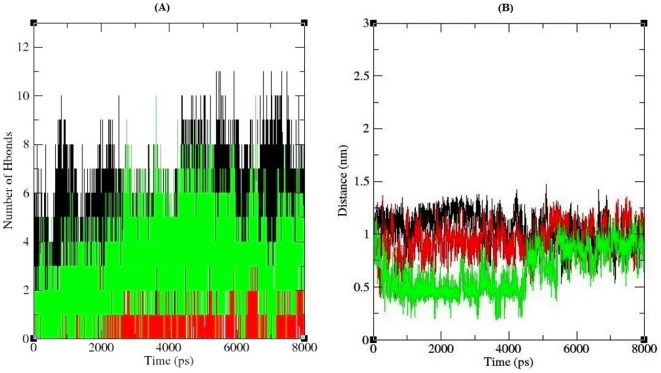
Inter molecular hydrogen bond and salt bridge formation of APE1-DNA complexes. (**A**) Number of hydrogen bond formed between APE1 protein and its binding DNA. Black, Red and Green lines indicate hydrogen bonds of the native, I64T and P311S structures respectively. (**B**) Stabilizing salt bridges formed between APE1 protein and DNA. The ordinate is distance (nm) and the abscissa is time (ps). Black, Red and Green lines indicate native, I64T and P311S complexes respectively.

**Figure 8:**
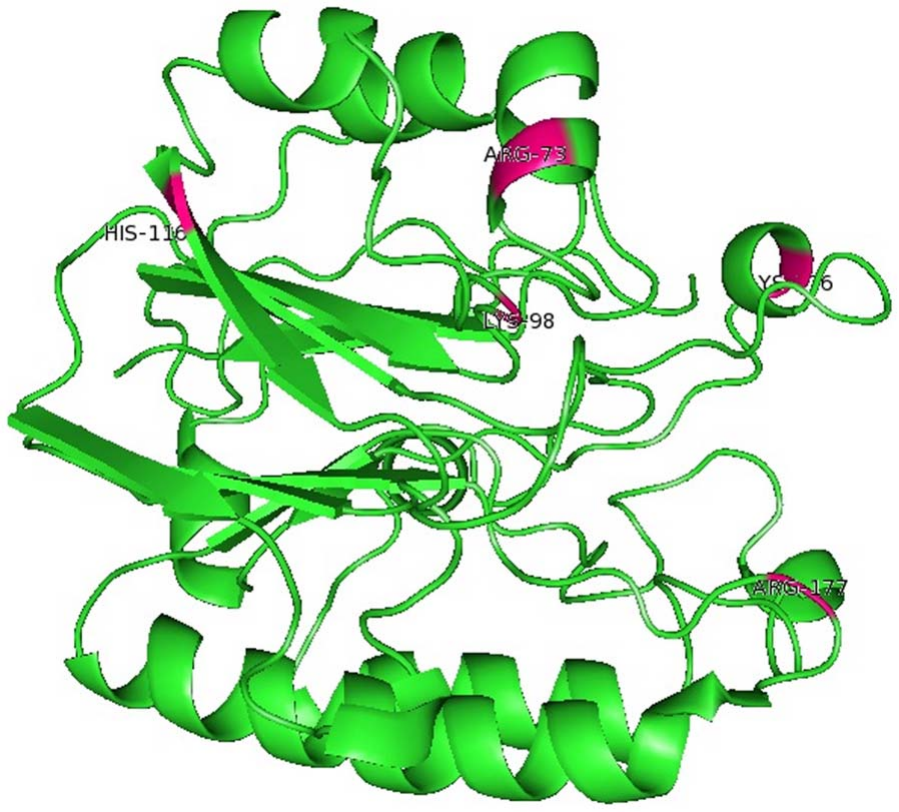
Functional residues of APE1 protein involved in salt bridge formation (ARG 73, LYS 98, HIS 116, ARG 177 and LYS 276) were labeled and colored as pink.

Hydrogen bonds are by far the most important specific interactions in biological recognition processes. Non synonymous SNPs can affect wild type protein function by affecting hydrogen bond formation between patterns [34]–[36]. Figure 7A depicts the number of hydrogen bonds formed between protein and DNA for native, I64T and P311S complexes. Native complex exhibited ∼2–11 number of hydrogen bonds throughout the simulation period, while the P311S showed higher number of hydrogen bonds ∼2–10 when compared to I64T complex of ∼2–5 hydrogen bonds in the entire simulation. These results infers that mutation might destroy the ability of hydrogen bond formation between protein and DNA, and this agreed with the stability of mutant models observed from RMSD and RMSF analysis. In additional to MD approach, intra molecular hydrogen bonds were analyzed using Hydrogen bond Calculation (Ver 1.1) server [37], [38]. GLY71, ARG73, ALA74, LYS98, GLU96, THR97, GLY127, TYR128, ARG156, TYR171, ASN174, ARG177, ASN226, ASN229, TYR269, LYS276, TRP280 and ASP 308 were the functional residues of APE1 protein which makes contact with DNA [3]. Among the 18 functional residues, except ASN174, ARG177 and ASP308, remaining 15 residues were involved in hydrogen bond formation with its neighboring residues (Table S1). It was observed that each hydrogen bond distance, and angle was changed in I64T and P311S while compare to native protein. Change in the hydrogen bond distance and angle may affect the stability of protein. In neutral solution, basic amino acids gain proton and become positively charged. Interaction between positive ions in protein and negative ions in DNA form salt bridge, which is an important stabilizing force. The presence of salt-bridge is an evidence of close proximity in the structure. Salt bridge formed between APE1 protein and DNA for all three trajectories were calculated and shown in Figure 7B. In the range ∼0–1.25 nm considerable distance deviation was observed between I64T and P311S, and shows greatest deviation from native complex. All three complexes reach the stable distance of ∼1 nm at the range of simulation period ∼5–8 ns. Up to ∼5 ns of simulation clear deviation in distance for I64T and P311S were observed and from ∼5 ns to the end point of simulation period limited deviation was observed. These results infer that mutation affects the salt bridge stability between protein and DNA. To strengthen our molecular dynamics approach, unbound native and mutant models were further analyzed in ESBRI server [39]–[42]. Intra molecular salt bridge analysis shows that among the eighteen functional residues five residues (ARG73, LYS98, HIS116, ARG177 and LYS276) were involved in salt bridge formation (Figure 8). ARG73 forms salt bridge with GLU101, ARG177 forms salt bridge with GLU96, ASP210 and ASP308, LYS276 makes salt bridge contact with GLU87, and LYS98 and HIS116 forms salt bridge with LYS276 respectively. Each contact distance was calculated and shown in Table 3. It was also noted that each salt bridge contact distance was changed in I64T and P311S, when compared to the native protein. From this analysis we infer that salt bridges are more stable in native state, while in the mutant state became weak in the presence of mutations.

**Table 3:** Salt bridge distances between positive and negative residues of APE1 protein native and mutant models.

Residue 1 and its position	Residue 2 and its position	Native Distance (nm)	T313A Distance (nm)	I64T Distance (nm)	G241R Distance (nm)	I64V Distance (nm)	P311S Distance (nm)	A317V Distance (nm)	D148E Distance (nm)	Q51H Distance (nm)
ARG 73	GLU101	3.87	3.14	3.75	3.17	3.37	3.67	3.37	3.54	3.73
LYS 98	ASP 70	3.15	3.54	3.17	3.51	3.87	3.22	3.55	3.56	3.22
HIS 116	ASP 70	3.24	3.16	3.23	2.55	2.78	2.48	3.45	3.67	2.45
ARG177	GLU 96	2.50	3.22	2.7	3.49	3.87	2.79	3.57	3.23	3.58
ARG177	ASP 210	3.87	3.43	3.85	3.56	3.65	3.83	3.17	3.67	3.90
ARG177	ASP 308	3.83	3.78	3.88	3.65	3.78	3.9	3.88	3.23	3.78
LYS276	GLU 87	2.47	3.18	2.44	2.45	2.54	3.19	3.56	3.67	3.67

**Figure 9:**
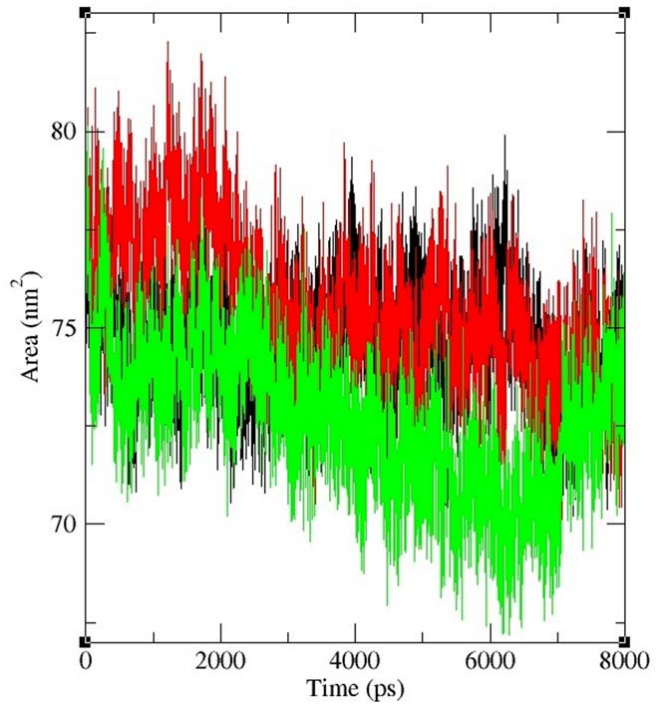
Solvent accessible surface area (SASA) of APE1 protein. Black, Red and Green lines indicate SASA of the native, I64T and P311S structures respectively. The ordinate is area (nm square) and the abscissa is time (ps).

The solvent accessible surface area (SASA) of a bimolecular is that, accessible to a solvent and it can be related to the hydrophobic core. It is typically calculated using the ‘rolling ball’ algorithm [43]. Solvent accessibility was divided predominantly into buried and exposed region, indicating the least accessibility and high accessibility of the amino acid residues to the solvent [44]. Solvent accessible area was calculated for native and mutant trajectories value and depicted in Figure 9. It is evident that native and P311S exhibited similar fashion of solvent accessible surface area ∼72–77 nm square on the dynamic period of ∼0–3 ns, while I64T displayed solvent accessible area of ∼75–82 nm square. In the rest of the dynamic period of ∼3–8 ns, inversely native and I64T displayed similar fashion of solvent accessible area ∼72–78 nm square. In contrast P311S displayed very low solvent accessible area of ∼67–76 nm square. Increase or decrease in the solvent accessible surface area indicates the change in exposed amino acid residues and it could affect the tertiary structure of the proteins.

## Discussion

Owing to the application of high-throughput sequencing technologies, the number of identified genomic variants especially SNPs in the human genome is growing rapidly. This has yielded massive amounts of data on human SNPs, and this information investigates human complex diseases such as cancer. It has been estimated that the NCBI contains ∼25 million human entries in the release of build 130 [29]. Between the different classes of SNPs, nsSNPs plays an important role at both functional and structural level in the encoded protein to cause disease. Discriminating the deleterious nsSNPs over neutral ones for specific phenotype by experimental method is labor intensive. *In silico* methods especially can aid in identifying disease-causing mutations by helping in the selection and prioritization of likely candidates from a pool of data. Most of the disease causing deleterious nsSNPs may lead to alterations in the structure, folding, or stability of the protein product, thereby altering or preventing the function of the protein. Yue and Moult [45] investigated the effect of nsSNPs on protein stability, and estimated that approximately 25% of nsSNPs in the human population might be deleterious to protein function. In another study by Wang and Moult reported that, the vast majority of the disease associated nsSNPs in their dataset (up to 80%) resulted in protein destabilization [25]. Several *in silico* methods have been developed to predict whether disease-related nsSNPs is deleterious or benign. Evolutionary information is commonly considered to be the most important feature for such a prediction task. In this analysis, we employed four widely-used computational tools SIFT, PolyPhen, I-Mutant 2.0 and PANTHER for determining the functional significance of nsSNPs in *APE1* gene. These methods differ in the properties of the variant they take into account in the prediction, as well as in the nature and possible training of the classification method used for decision making. All the variation tolerance methods analyzed in this study follow a similar procedure in which a missense variant is first labeled with properties related to the damage it may cause to the protein structure or function. The basis for predicting the impact of mutations in these four algorithms are different and we would expect that the outcomes to be in some ways, dissimilar. However, the mutations that overlap the four predictions should provide greatest reliability to behave similarly.

Deleterious nsSNPs can alter normal protein function, by means of geometric constraint changes [46], hydrophobic changes [47], [48], and disruption of salt bridges or hydrogen bonds [47], [49]. Sunyaev et al. and Chen et al. also indicated that the residue solvent accessibility, which could identify the buried residues, was confidently proposed as predictors of deleterious substitutions [50], [51]. Xi et al. and, Yu and Hadi in their analysis characterized the deleterious nsSNPs in *APE1* gene using *in silico* tools such as SIFT and PolyPhen [15], [16]. Also molecular dynamic study was carried out by Abyzov et al. to evaluate the interactions between APE1and pol β protein complexes with DNA molecule [52]. In this study, we tried to evaluate the deleterious nsSNPs at structural level in three contexts: (1) identifying deleterious nsSNPs by both sequence and structure based methods (2) changes in protein stability by DDG score (3) measuring alterations of protein 3D structure by deleterious nsSNPs by molecular dynamics approach.

By applying all four *in silico* tools, we identified two nsSNPs I64T and P311S as highly deleterious based on the scores. To better understand how these deleterious nsSNPs affect the structural behavior of *APE1* gene, we incorporated molecular dynamic approach using GROMACS force field 43a1. On 8ns simulation trajectory, different parameters were applied to investigate the molecular behavior of native and mutant (I64T and P311S) complexes. Structural validations for all three complexes were done by RMSD and RMSF analysis. RMSD analysis results inferred that native complex reach final deviations of ∼0.2 nm while the mutant complexes I64T and P311S showed high deviation value of ∼0.25 nm. In RMSF analysis, I64T and P311S shows maximum fluctuation value of ∼0.35 nm on the residue range of ∼110 and ∼27 respectively, on other hand native complex have low fluctuation value ∼0.1 nm and ∼0.25 nm on the same residue range. Based on RMSD and RMSF analysis, we confirmed that I64T and P311S showed different path of deviation and fluctuation to I64T and P311S complexes, which in turn leads to conformational change in mutant complexes. Structural mutations were found to affect buried residues in the protein core, causing changes in amino acid size, amino acid charge, hydrogen bonds, salt bridges, S-S bridges [53]. In order to investigate the effect of structural mutations in functional changes, electrostatic interaction analyses were performed between protein and DNA molecules of both native and mutant complexes. Hydrogen bonds and salt bridges play a central role in protein to make stable contact with its binding target. In addition, as early studies have shown, protein active sites often provide electrostatic complementarity to the charge distribution of the binding substrates [54]–[57]. Taken together, mutation may change the electrostatic states and affect the binding interfaces tend to form more or less hydrogen bonds and salt bridges than native protein. Accordingly in average 3, 8 and 11 numbers of hydrogen bonds were observed between APE1 and its binding DNA in I64T, P311S and native complexes respectively. The mean number of total H-bonds during the entire simulation differs drastically between the native and mutant models. Since the difference is great, the reduced number of H-bonds may affect the binding stability. In salt bridge analysis it was observed that different pattern of distance maintained by I64T and P311S complexes. Even though the mutated proteins maintain salt bridge distance ≤4.0 Å with DNA, they show different pattern of distance while compared to the distance maintained by native protein. It reflects that the modification occurred in the cationic side chains residues of mutant proteins. In both hydrogen bond and salt bridge analysis of unbound protein, bonding distance changes was observed. These changes in distance have been shown to cause loss of thermodynamic stability as well as aberrant folding and aggregation of the protein [53]. These changes may modify APE1 structure which leads to affect the binding facility of APE1 protein. Furthermore in SASA analysis, it was found that with respect to native, unequal area of solvent accessible surface observed in two mutant modeled proteins. P311S show very less and I64T show intermediate accessible area while compared to native protein. Less the accessible area decrease the probability of interaction with molecules. Thus SASA analysis elucidates that presence of deleterious mutations in APE1 residues results changes in hydrophilic area of mutant proteins. From our simulation analysis we reported that the predicted deleterious nsSNPs (I64T and P311S) in *APE1* gene could affect APE1 protein natural behavior which may be used as suitable bio markers for detecting people at risk for certain diseases.

## Materials and Methods

### Dataset

The protein sequence for *APE1* gene was obtained from SWISS-Prot database [31]. The SNPs and their relevant information of *APE1* gene were retrieved from National Centre for Biotechnology Information (NCBI) database [29] and the 3D structure of APE1-DNA complex was obtained from PDB database [58].

### Prediction of deleterious variants

Each nucleotide substitution has the potential to affect protein function. We used two diverse approaches, Empirical and Support Vector Machine (SVM) to determine functional SNPs in *APE1* gene. Sequence (SIFT, PANTHER) and structure based methods (PolyPhen, I-Mutant 2.0) are the most common approaches used in SNP prediction tools. SIFT, PANTHER and I-mutant give results in two prediction categories-tolerated or deleterious effects, while PolyPhen gives results in three categories-benign (probably lacking any phenotypic effect), possibly damaging, and probably damaging (should affect protein function). Sequence-based prediction includes all types of effect at the protein sequence level and can be applied to any human protein with known relatives. Structure-based approach is feasible to implement for the proteins with 3D structures. Tools that integrate both sequence and structure information have the added advantage of being able to assess the reliability of the generated prediction results by cross-referencing the results from both approaches. Tools that combine these approaches use different algorithms and methodologies for prediction, thereby having a wider cover-age of the different aspects of SNP analysis. SIFT predicts whether an amino acid substitution affects protein function based on sequence homology and the physical properties of amino acids [21]. SIFT score ≤0.05 indicates the amino acid substitution is intolerant or deleterious, where as score ≥0.05 is predicted to be tolerant [59], [60]. PANTHER estimates the likelihood of a particular nsSNP causing a functional impact on the protein [22]. PANTHER uses HMM-based statistical modeling methods and multiple sequence alignments to perform evolutionary analysis of coding nsSNPs. PANTHER subPSEC scores vary from 0 (neutral) to about −10 (most likely to be deleterious). Protein sequences having subPSEC value≤−3 is said to be deleterious. PolyPhen uses sequence, phylogenetic and structural information in characterizing the deleterious substitution. A PSIC score difference of 1.5 and above is considered to be damaging. I-Mutant 2.0 [23] is a SVM-based method for the automatic prediction of protein stability changes upon single point mutations. The output file shows the predicted free energy change (DDG) which is calculated from the unfolding Gibbs free energy change of the mutated protein minus the unfolding free energy value of the native protein (Kcal/mol). DDG>0 means that the mutated protein has high stability and vice versa.

### Molecular Dynamics Simulation protocol

Molecular mechanics potential energy minimization and MD simulations were carried out using the program package GROMACS 4.0.5 [61], [62]. Force field GROMOS96 [63] 43a1 was used in all MD simulations. GROMACS have the limitation to parameterize heteroatom group in PDB file. To include heteroatom's, topology file was prepared by using PRODRG [64] server. Energy minimized structures of native APE1-DNA complex and two mutants were used as a starting point for MD simulations. The Protein-DNA complex was solvated in a cubic 0.9 nm of simple point charge (SPC) water molecules [65]. A periodic boundary condition was applied that the number of particles, pressure, and temperature was kept constant in the system. The system was neutralized by adding chlorine ions around the molecule. In this step one Cl^−^ ion was added to both native and mutant structures. It results in substitution of random water molecule with one chlorine ion in order to obtain neutralized system. The temperature was kept constant by using Berendsen thermostat [66] with a coupling time of 0.2 ps. All Protein-DNA complex atoms were at a distance equal to 1.0 nm from the box edges. The minimized system was equilibrated for 1000 ps each at 300 K by position restrained molecular dynamics simulation in order to soak the water molecules in to the macromolecules. The equilibrated systems were then subjected to molecular dynamics simulations for 8 ns each at 300 K. In all simulations, the temperature was kept constant at 300 K. The particle mesh Ewald method [67] was used to treat long-range Coulombic interactions and the simulations performed using the SANDER module [68]. SHAKE algorithm was used to constrain bond lengths involving hydrogen's, permitting a time step of 2 fs. Van der Waals force was maintained at 1.4 nm and coulomb interactions were truncated at 0.9 nm.

### Trajectory Analysis

The trajectory files were analyzed through g_rmsd and g_rmsf GROMACS utilities in order to obtain the root-mean-square deviation (RMSD) and root-mean square fluctuation (RMSF) values. Number of distinct hydrogen bonds formed between protein and DNA during these simulations were calculated using g_hbond utility. Number of hydrogen bonds determined on the basis of donor-acceptor distance smaller than 3.9 nm and of donor-hydrogen-acceptor angle larger than 90 nm [69]. Intra molecular hydrogen bond distance and angle was calculated by “Hydrogen Bond Calculation” server. Salt bridge formed between protein, and DNA was analyzed by using g_salt GROMACS utility and intrarmolecular salt bridge analysis was performed by ESBRI (Evaluating the Salt BRIdges in protein) server [37]–[40]. If the distance is ≤4.0 nm the pair is counted as a salt bridge [70]. Further, SASA calculated by g_sas utility. In order to generate the 3D backbone RMSD, RMSF of carbon-alpha, hydrogen bonds, salt bridges, SASA analysis and motion projection of the protein in phase space of the systems were plotted for all three simulations using the GRaphing, Advanced Computation and Exploration (GRACE) program.

## Supporting Information

Table S1 **Summary of intra-molecular hydrogen bonds formation in native and mutant models of APE1 protein.**
(DOC)
