# Supplementary material for: Investigating the Structural Impacts of I64T and P311S Mutations in APE1-DNA Complex: A Molecular Dynamics Approach
Source: PLoS One. 2012 Feb 27;7(2):e31677. doi: 10.1371/journal.pone.0031677 (PMC3288039; doi:10.1371/journal.pone.0031677)
Supplement: Table S1 — Summary of intra-molecular hydrogen bonds formation in native and mutant models of APE1 protein. (DOC) [file pone.0031677.s001.doc]

**Table S1. Summary of intra-molecular hydrogen bonds formation in native and mutant models of APE1 protein.**

| **Residue 1** | **Residue 2** | **Native H-bond length** | **T313A H-bond length** | **I64T H-bond length** | **G241R H-bond length** | **I64V H-bond length** | **P311S H-bond length** | **A317V H-bond length** | **D148E H-bond length** | **Q51H H-bond length** | **Native H-bond angle** | **T313A H-bond angle** | **I64T H-bond angle** | **G241R H-bond angle** | **I64V H-bond angle** | **P311S H-bond angle** | **A317V H-bond angle** | **D148E H-bond angle** | **Q51H H-bond angle** |
| --- | --- | --- | --- | --- | --- | --- | --- | --- | --- | --- | --- | --- | --- | --- | --- | --- | --- | --- | --- |
| GLY 71 | GLU 96 | **1.86** | 1.94 | 1.90 | 1.94 | 1.94 | 1.92 | 1.94 | 1.94 | 1.94 | **161.53** | 163.88 | 161.47 | 163.88 | 163.88 | 161.72 | 163.88 | 163.88 | 163.88 |
| GLY 71 | TRP 75 | **2.15** | 2.22 | 2.15 | 2.22 | 2.22 | 2.14 | 2.22 | 2.22 | 2.22 | **149.37** | 135.84 | 149.35 | 135.84 | 135.84 | 150.19 | 135.84 | 135.84 | 135.84 |
| ARG 73 | GLU101 | **3.27** | 2.91 | 3.15 | 2.43 | 2.43 | 3.10 | 2.91 | 2.91 | 3.23 | **115.86** | 123.82 | 114.66 | 101.18 | 101.18 | 113.65 | 123.82 | 123.82 | 112.13 |
| ARG 73 | LYS 98 | **3.33** | 2.02 | 3.22 | 2.02 | 2.02 | 3.17 | 2.43 | 2.32 | 2.02 | **148.22** | 143.17 | 147.22 | 160.62 | 160.62 | 146.55 | 149.35 | 149.35 | 160.62 |
| ARG 73 | LYS 77 | **1.92** | 2.02 | 1.93 | 2.91 | 2.91 | 1.93 | 2.02 | 2.02 | 2.91 | **156.03** | 160.62 | 155.52 | 123.82 | 123.82 | 155.42 | 160.62 | 160.62 | 123.82 |
| ALA 74 | LYS 78 | **1.94** | 2.36 | 1.89 | 2.36 | 2.36 | 1.87 | 2.36 | 2.36 | 2.36 | **148.80** | 132.99 | 149.74 | 132.99 | 132.99 | 150.40 | 132.99 | 132.99 | 132.99 |
| LYS 78 | ALA 74 | **1.94** | 2.36 | 1.89 | 2.36 | 2.36 | 1.87 | 2.36 | 2.36 | 2.36 | **148.80** | 132.99 | 149.74 | 132.99 | 132.99 | 150.40 | 132.99 | 132.99 | 132.99 |
| LYS 78 | GLY306 | **2.92** | 2.02 | 2.82 | 1.56 | 1.88 | 2.81 | 2.43 | 2.02 | 1.90 | **125.28** | 150.40 | 127.50 | 132.78 | 145.41 | 126.43 | 143.17 | 149.35 | 114.61 |
| GLU 96 | ASN 68 | **2.98** | 2.78 | 3.06 | 2.89 | 1.94 | 3.12 | 2.78 | 2.78 | 2.78 | **116.29** | 106.09 | 117.53 | 151.44 | 163.88 | 116.86 | 106.09 | 106.09 | 106.09 |
| GLU 96 | GLY 71 | **1.86** | 1.94 | 1.90 | 1.94 | 2.78 | 1.92 | 1.94 | 1.94 | 1.94 | **161.53** | 163.88 | 161.47 | 163.88 | 106.09 | 161.72 | 163.88 | 163.88 | 163.88 |
| THR 97 | GLY130 | **1.76** | 1.75 | 1.77 | 1.75 | 1.75 | 1.77 | 1.75 | 1.75 | 1.75 | **127.97** | 121.08 | 126.55 | 121.08 | 121.08 | 125.60 | 121.08 | 121.08 | 121.08 |
| THR 97 | LEU72 | **1.81** | 1.84 | 1.82 | 1.84 | 1.75 | 1.82 | 1.84 | 1.84 | 1.84 | **123.70** | 131.29 | 123.20 | 131.29 | 121.08 | 123.19 | 131.29 | 131.29 | 131.29 |
| GLY127 | SER100 | **2.05** | 1.67 | 2.05 | 2.22 | 1.67 | 2.04 | 1.67 | 1.67 | 1.67 | **160.67** | 172.38 | 156.74 | 156.87 | 172.38 | 155.77 | 172.38 | 172.38 | 172.38 |
| TYR128 | GLU154 | **2.73** | 2.99 | 2.65 | 2.08 | 2.99 | 2.64 | 2.99 | 2.99 | 2.99 | **108.16** | 121.54 | 108.23 | 143.17 | 121.54 | 108.54 | 121.54 | 121.54 | 121.54 |
| TYR128 | ARG156 | **3.02** | 2.32 | 3.04 | 2.08 | 2.99 | 3.05 | 2.99 | 1.96 | 2.99 | **112.03** | 149.35 | 109.29 | 145.08 | 145.08 | 108.09 | 121.54 | 149.22 | 121.54 |
| TYR128 | GLU154 | **2.73** | 2.02 | 2.81 | 2.08 | 2.55 | 2.64 | 2.43 | 2.97 | 2.55 | **140.68** | 150.40 | 122.14 | 154.08 | 144.08 | 137.49 | 143.17 | 166.97 | 144.08 |
| ARG156 | TYR171 | **2.81** | 2.17 | 3.07 | 2.35 | 2.17 | 2.81 | 2.17 | 2.17 | 2.17 | **122.83** | 132.95 | 157.18 | 102.16 | 132.95 | 121.82 | 132.95 | 132.95 | 132.95 |
| ARG156 | GLU154 | **3.04** | 2.32 | 3.04 | 2.64 | 2.32 | 3.08 | 2.32 | 2.32 | 2.32 | **156.14** | 125.91 | 109.29 | 113.18 | 125.91 | 158.32 | 125.91 | 125.91 | 25.91 |
| ARG156 | TYR128 | **3.02** | 2.73 | 1.98 | 2.78 | 2.99 | 3.05 | 2.99 | 2.99 | 2.73 | **112.03** | 137.18 | 142.75 | 173.17 | 121.54 | 108.09 | 121.54 | 121.54 | 137.18 |
| ARG156 | TYR171 | **1.99** | 2.99 | 1.98 | 2.56 | 2.99 | 1.97 | 2.73 | 2.73 | 2.99 | **141.97** | 168.58 | 142.75 | 168.58 | 121.54 | 143.17 | 137.18 | 137.18 | 156.77 |
| TYR171 | ARG156 | **1.99** | 2.17 | 2.81 | 2.57 | 2.17 | 1.97 | 2.17 | 2.17 | 2.65 | **141.97** | 132.95 | 122.14 | 146.25 | 132.95 | 143.17 | 132.95 | 132.95 | 167.98 |
| TYR171 | ARG156 | **2.81** | 2.73 | 2.01 | 2.32 | 2.73 | 2.81 | 2.73 | 2.73 | 2.17 | **122.83** | 137.18 | 129.18 | 137.18 | 132.95 | 121.82 | 137.18 | 137.18 | 25.91 |
| ASN226 | PRO223 | **2.02** | 2.50 | 2.03 | 2.47 | 2.50 | 2.01 | 2.50 | 2.50 | 2.50 | **129.49** | 107.86 | 121.33 | 109.79 | 107.86 | 128.90 | 107.86 | 107.86 | 107.86 |
| ASN226 | ASN229 | **2.00** | 1.80 | 2.03 | 2.47 | 1.80 | 2.04 | 1.80 | 1.80 | 2.50 | **119.89** | 111.43 | 121.33 | 173.17 | 111.43 | 122.08 | 111.43 | 111.43 | 107.86 |
| ASN229 | ASN226 | **2.00** | 1.80 | 2.23 | 2.67 | 1.80 | 2.04 | 1.80 | 1.80 | 1.80 | **119.89** | 111.43 | 139.77 | 163.42 | 111.43 | 122.08 | 111.43 | 111.43 | 111.43 |
| ASN229 | PHE232 | **2.26** | 2.33 | 3.15 | 2.65 | 2.33 | 2.22 | 2.33 | 2.33 | 2.33 | **139.33** | 142.50 | 102.99 | 138.76 | 142.50 | 140.06 | 142.50 | 142.50 | 142.50 |
| TYR269 | ASP 70 | **3.31** | 2.32 | 3.15 | 1.23 | 1.56 | 3.05 | 2.43 | 2.32 | 2.33 | **100.90** | 137.18 | 136.71 | 149.80 | 176.34 | 104.38 | 143.17 | 137.18 | 156.67 |
| TYR269 | ASP 70 | **3.31** | 2.32 | 1.96 | 2.92 | 1.78 | 3.05 | 2.32 | 2.32 | 2.33 | **136.94** | 143.17 | 146.96 | 173.17 | 145.93 | 137.03 | 137.18 | 149.35 | 142.50 |
| VAL278 | ALA273 | **1.96** | 1.99 | 1.88 | 2.29 | 1.99 | 1.96 | 1.99 | 1.99 | 1.99 | **147.44** | 149.37 | 144.43 | 143.87 | 149.37 | 146.33 | 149.37 | 149.37 | 149.37 |
| TRP 280 | PHE266 | **1.89** | 1.88 | 2.02 | 2.68 | 1.88 | 1.87 | 1.88 | 1.88 | 1.88 | **142.44** | 127.22 | 133.46 | 198.49 | 127.22 | 145.40 | 127.22 | 127.22 | 127.22 |
| TRP 280 | THR265 | **2.01** | 1.75 | 1.85 | 1.23 | 1.75 | 2.03 | 1.75 | 1.75 | 1.75 | **133.10** | 130.35 | 160.04 | 163.42 | 130.35 | 133.25 | 130.35 | 137.18 | 130.35 |
| TRP 280 | PHE266 | **1.83** | 1.72 | 2.45 | 2.92 | 1.72 | 1.85 | 1.72 | 1.72 | 1.72 | **160.93** | 163.07 | 112.79 | 138.76 | 163.07 | 159.05 | 163.07 | 163.07 | 163.07 |

**Native protein H- bond length and H-bond angle were highlighted in bold.**
